# Practical Aspects of microRNA Target Prediction

**DOI:** 10.2174/156652411794859250

**Published:** 2011-03

**Authors:** T.M Witkos, E Koscianska, W.J Krzyzosiak

**Affiliations:** Laboratory of Cancer Genetics, Institute of Bioorganic Chemistry, Polish Academy of Sciences, Noskowskiego 12/14 Str., 61-704 Poznan, Poland

**Keywords:** Bioinformatics, gene regulation, miRNA, miRNA-mRNA interaction, neurodegeneration, target prediction algorithm, target validation, trinucleotide repeat expansion disorders.

## Abstract

microRNAs (miRNAs) are endogenous non-coding RNAs that control gene expression at the posttranscriptional level. These small regulatory molecules play a key role in the majority of biological processes and their expression is also tightly regulated. Both the deregulation of genes controlled by miRNAs and the altered miRNA expression have been linked to many disorders, including cancer, cardiovascular, metabolic and neurodegenerative diseases. Therefore, it is of particular interest to reliably predict potential miRNA targets which might be involved in these diseases. However, interactions between miRNAs and their targets are complex and very often there are numerous putative miRNA recognition sites in mRNAs. Many miRNA targets have been computationally predicted but only a limited number of these were experimentally validated. Although a variety of miRNA target prediction algorithms are available, results of their application are often inconsistent. Hence, finding a functional miRNA target is still a challenging task. In this review, currently available and frequently used computational tools for miRNA target prediction, i.e., PicTar, TargetScan, DIANA-microT, miRanda, rna22 and PITA are outlined and various practical aspects of miRNA target analysis are extensively discussed. Moreover, the performance of three algorithms (PicTar, TargetScan and DIANA-microT) is both demonstrated and evaluated by performing an in-depth analysis of miRNA interactions with mRNAs derived from genes triggering hereditary neurological disorders known as trinucleotide repeat expansion diseases (TREDs), such as Huntington’s disease (HD), a number of spinocerebellar ataxias (SCAs), and myotonic dystrophy type 1 (DM1).

## INTRODUCTION

microRNAs (miRNAs) are small non-coding RNAs about 22 nucleotides in length that play an important role in posttranscriptional regulation of target genes both in plant and animal cells. It is estimated that over half of mammalian protein coding-genes are regulated by miRNAs and most human mRNAs have binding sites for miRNAs [1, 2]. Until now 1048 human miRNAs have been annotated in miRNA registry (miRBase, Release 16) [3] and this number is steadily growing. miRNAs downregulate gene expression mostly by imperfect binding to complementary sites within transcript sequences and suppress their translation, stimulate their deadenylation and degradation or induce their cleavage [4, 5]. miRNAs originate from genome-encoded primary transcripts (pri-miRNAs) forming distinctive hairpin structures that are cleaved by the ribonuclease Drosha [6, 7] to ~60 nucleotide-long pre-miRNAs which are further trimmed by the ribonuclease Dicer [8, 9] to imperfect ~22-nt duplexes. One strand of the duplex is incorporated into RNA-induced silencing complex (RISC) [10, 11] and becomes a functional miRNA. miRNAs have been shown to be crucial for the majority of physiological processes; development, cell proliferation and cell death [12, 13]. Therefore, their deregulation has been associated with many diseases such as cancer, cardiovascular and neurodegenerative diseases or metabolic disorders [14-17]. Hence, miRNA expression analysis has a diagnostic value and normalizing miRNA levels is one of the promising therapeutic approaches [18-20].

Several types of miRNA target binding sites, differing in the position and localization of Watson-Crick pairings and mismatches, have been distinguished [21]. Plant miRNA target sites are located within open reading frames (ORFs) of target genes and nearly full complementarity is required between miRNAs and their target transcripts [22]. Therefore, a basic matching of plant miRNA and mRNA sequences serves as an efficient and accurate prediction method [23]. Unfortunately, this strategy could not be simply applied to the animal models since strict complementarity between the target site and the miRNA is a rare phenomenon [24]. Moreover, miRNAs are predominantly found in 3’ untranslated regions (3’ UTRs) of target genes and only sporadically in 5’UTRs or ORFs [25-27].

Effective prediction of miRNA-mRNA interactions in animal systems remains challenging due to the interaction complexity and a limited knowledge of rules governing these processes. Therefore, it is necessary to take advantage of the newest findings in miRNA biology and their targets prediction algorithms to find possible miRNA-mRNA interactions. Numerous target prediction algorithms exploiting different approaches have been recently developed, and many methods of experimental validation have been designed. In this review we summarize hitherto known facts on miRNA-mRNA interactions, describe functions and approaches of commonly used target predictions algorithms and methods for experimental validation. We also draw attention to practical aspects of computational miRNA target analysis and present our approach to miRNA target prediction using the example of genes causing trinucleotide repeat expansion diseases (TREDs).

## miRNA-mRNA INTERACTIONS

**Fig. (1). Types of miRNA-mRNA interactions.**
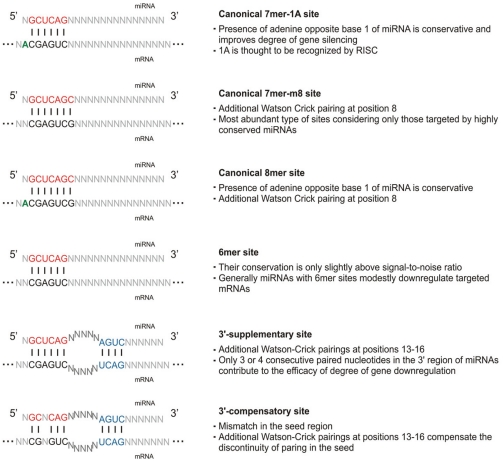
Different classes of miRNA target sites are presented in a schematic way. Vertical dashes represent single Watson-Crick pairing. Nucleotides involved in binding have been arbitrarily defined to depict positions of required complementarity between miRNA and mRNA. Seed regions of miRNAs are marked by red color and the adenine at binding position 1 by green. Interactions between mRNA and the 3’ end of miRNA have not been shown because they are sequence-dependent and do not significantly contribute to the miRNA downregulation effect. In the case of 3’-suppelmentary and 3’-compensatory sites two regions of pairing (base pairs colored in blue) force middle mismatches to form a loop structure. Additionally, features of particular site types have been listed.

Target sites for animal miRNAs are not evenly distributed within 3’UTR but they rather tend to group at both ends of 3’UTR [28]. It is typical of genes with short 3’UTRs to have target sites at 5’ part of 3’UTR [29]. Alternative transcripts with varied length of 3’UTRs could be regulated by different sets of miRNAs [30]. There is also an abundance of mRNAs having potential multiple sites for the same miRNA [31-33]. It was reported that multiple sites enhance the degree of downregulation [34] and many target prediction algorithms exploit this fact in their search and scoring. Two sites of the same or different miRNAs located closely to each other could act synergistically [33, 35]. Regions of strict complementarity, bulges and mismatches have been observed in almost all known miRNA-mRNA interactions in animals [21, 36]. Each of these duplexes has a region called the “seed”, a site located at 5’ part of miRNA (positions 2-7) which is characterized by a strict or almost strict Watson-Crick pairing between miRNA and its target site and shows the best conservation among the miRNA sequence [37]. It has to be pointed out that there is no single model that would depict all miRNA-mRNA interactions because of their relative heterogeneity. The classification of miRNA target sites is based on the complementarity within 5’ (the seed region) and 3’ part of miRNA (Fig. **1**) and distinguishes three types of sites: 1) canonical, 2) 3’-supplementary and 3) 3’-compensatory sites [21]. 

The majority of known targets called canonical sites have a complete paring within the seed region which determines the certainty of the interaction. There are three types of canonical sites [38]: the 7mer1A that has an adenine in position 1 at the 5’ end of miRNA, the 8mer having matched adenine in position 1 and an additional match in position 8 and the 7mer-m8 that has a match in position 8. Canonical sites account for the majority of validated conserved targets, 7mer sites are most abundant for highly conserved miRNAs [1] and the adenine opposite position 1 of miRNA improves the degree of gene silencing [39]. There are also known sites with shorter, 6-nt seed but they are thought to have a limited functionality. All of these groups can have an additional pairing within 3’ part of miRNA and corresponding nucleotides of the transcript (3’-supplementary sites) but it usually has a less profound effect on target recognition and its efficacy [33]. At least 3-4 nucleotides consecutively paired in positions 13-16 of miRNA are usually required to enhance the effectiveness of miRNA-mRNA interaction which facilitates target prediction. There is also a possibility of a mismatch in the seed which is compensated by additional extended pairing in 3’ part of miRNA (3’-compensatory sites).

The complexity of miRNA-mRNA interactions is one of the main reasons why algorithms based on a miRNA-mRNA sequence matching only are insufficient and additional parameters such as orthologous sequences alignment, UTR context or free energy of complexes have to be taken into account. 3’UTR orthologous sequence analysis is a basic method of making the miRNA target prediction more efficient [40] and it is restricted to a comparison of conservative sequences in human transcriptome to relatively evolutionally distant species such as the mouse, the dog or the fish. It is based on the assumption that sites, as targets for miRNA regulation, have been kept unchanged because of evolutionary pressure [1, 41]. It has been well proven that some miRNA families are highly conserved among closely related species, have many conserved targets and family members differ in the 3’ region that enables distinction of sensitivity to transcript targets [38]. Friedman *et al.* [1] suggested that most mammalian targets retain sites for conserved miRNAs. The use of an approach based on sequence conservation seems to be entirely justifiable for an analysis of seed regions [38, 42]. However, this strategy should be applied with caution because even conserved 3’UTRs have a large number of non-conserved targets. This is one of the main reasons why algorithms based on orthologous sequence alignment generate a number of false negative results. There is also an abundance of miRNAs that are not conserved and different approach is needed for prediction of their targets. In this case it is extremely important to implement other search parameters. It is known that simple base pairing is insufficient for miRNA target prediction [43] and the secondary structure of miRNA/target duplexes is a factor that should be taken into consideration [44, 45]. Many existing algorithms based on conservation analyses include the sequence-based binding energy of miRNA-target duplex calculations into a final score. Kertesz *et al.* [46] conducted a more in-depth analysis which centered on the site accessibility for miRNA downregulation efficacy. It turned out that upstream and downstream flank regions of miRNA binding sites tend to have weak base-pairing to reduce the energy cost of unpairing bases in order to make the site more accessible for RISC. This observation is in agreement with the fact that target flanking regions have high local AU content [33]. It was proposed that some miRNAs downregulate moderately targeted mRNAs and they are engaged in tuning gene-expression levels [47]. Moreover, certain target sites may act as competitive inhibitors of miRNA activity since the effect of miRNA regulation on them is very mild [48]. These sites will retain their conservation since they have a biological function as miRNA sequestration regulators.

## miRNA TARGET PREDICTION ALGORITHMS

Many different algorithms have been developed for prediction of miRNA-mRNA interactions. The rules for targeting transcripts by miRNAs have not been fully examined yet and are based mainly on experimentally validated miRNA-mRNA interactions [49, 50] that are only a slice of possibly existing *in vivo*. This situation led to the development of a variety of approaches to miRNA target prediction. The available algorithms have been extensively discussed by others [51-54]. Moreover, they were recently reviewed by Yue *et al.* [55] with the focus on their bioinformatical, mathematical and statistical aspects. The available algorithms can be classified into two categories established on the basis of the use or non-use of conservation comparison, a feature that influence greatly an outcome list of targets by narrowing the results [1, 33]. The algorithms based on conservation criteria are for example the following: miRanda [56], PicTar [42, 57], TargetScan [38], DIANA-microT [36]; while PITA [46] and rna22 [58] belong to the algorithms using other parameters, such as free energy of binding or secondary structures of 3’UTRs that can promote or prevent miRNA binding. Since all these algorithms were successfully used to predict miRNA targets in mammals we describe them in more detail below. Additionally, to facilitate the assessment of these algorithms, we summarize their performance and characteristic features (Table **1**).

### miRanda

The miRanda algorithm [56] is based on a comparison of miRNAs complementarity to 3’UTR regions. The binding energy of the duplex structure, evolutionary conservation of the whole target site and its position within 3’UTR are calculated and account for a final result which is a weighted sum of match and mismatch scores for base pairs and gap penalties. There is one wobble pairing allowed in the seed region that is compensated by matches in the 3’ end of miRNA. The usage of this strategy incorporates different nature of miRNA-mRNA interactions (Fig. **1**). miRNAs with multiple binding sites within 3’UTR are promoted, which contributes to the increase in specificity but it underestimates miRNAs with a single but perfect base pairing. It takes into account the evolutionary relationships of interactions more globally focusing on the conservation of miRNAs, relevant parts of mRNA sequences and the presence of a homologous miRNA-binding site on the mRNA [59].

### TargetScan and TargetScanS

These algorithms [38] use different approach to the prediction of interactions of miRNAs with mRNAs. Firstly, the search is narrowed to the sites that have full complementarity in the miRNA seed region that is defined as 6-nt long (nucleotides 2-7) and then they are extended to 21-23 nucleotide-long fragments representing true interactions. Results are classified within three groups on the basis of length of exact matching and an occurrence of adenine at the first position of mRNA target site which seems to be evolutionally conserved [39] and may act as a recognizing anchor for RISC. Several parameters determined in previous signal-to-noise analyses on an experimentally validated dataset contribute to the outcome score such as the type of seed matching, pairing contribution outside the seed region, AU content 30 nt upstream and downstream of predicted site and the distance to the nearest end of the annotated UTR of the target gene [33]. The conservation of seed regions among orthologous 3’UTRs within miRNA binding regions has a fundamental importance for an outcome score [1]. Less conservative miRNA-mRNAs interactions with wobble pairings and bulges, especially within 5’ region of miRNA, are also predicted in the newest versions.

TargetScanS [38] is an alternative, simplified version of TargetScan which predicts targets having a conserved 6-nt seed match flanked by either a 7-nt match or 6-nt with A on the 3’ terminus with no consideration of free energy values.

### PicTar

PicTar [42, 57] searches for nearly but not fully complementary regions of conservative 3’UTRs and then calculates the free energy of created duplexes. Each result is scored using Hidden-Markov Model (HMM – a simple example of dynamic Bayesian network), miRNAs with multiple alignments are favored. It was the first method that considered a parallel expression on the cellular level or an action in a common biological pathways of a miRNA and transcript. PicTar uses sequence alignment to eight vertebrate species to eliminate false positive results and it scores the candidate genes of each species separately to create a combined score for a gene. It is necessary for the mRNA to have recurring nucleobases at overlapping positions among species paired.

### DIANA-microT

This algorithm [36] uses a 38nt-long frame that is moved along 3’UTR. The minimum energy of potential miRNA binding, that allows mismatches, is measured after every shift and compares with the energy of 100 per cent complementary sequence bound to the 3’UTR region. DIANA-microT searches for sites with canonical central bulge and it requires 7, 8 or 9 nt–long complementarity in 5’ region of miRNA. 6 nt-long matches within seed region or with one wobble pairing are also considered while enhanced by additional base pairing in 3’ region of miRNA [60]. Because of exploiting experimental deduction of rules governing miRNA-mRNA site by their mutation it is constructed for single site prediction. DIANA-mciroT use conservative alignment for scoring but also considers non-conservative sites. It gives unique signal-to-noise ratio (SNR) which is a ratio between a total of predicted targets by single miRNA in searched 3’UTR and a total of predicted targets by artificial miRNA with randomized sequence in searched 3’UTR. It also provides users with a percentage probability of existence for each result depending on its pairing and conservation profile.

### PITA

PITA [46] offers a brand new view on the miRNA target prediction. It focuses on the target accessibility that is strictly connected to the secondary structure of the transcript. The main assumption is based on the fact that the mRNA structure plays a role in target recognition by thermodynamically promoting or disfavoring the interaction. PITA first predicts targets using complementarity analysis within seed regions (single mismatch or G:U wobble pairing can be allowed) and then compares the free energy gained from the formation of the miRNA-target duplex and the energetic cost of unpairing the target to make it accessible to the miRNA.

### Rna22

Rna22 [58] is a target prediction algorithm that is based on a search for patterns that are statistically significant miRNA motifs created after a sequence analysis of known mature miRNAs. It first searches for reverse complement sites of patterns within mRNA of interest and determines sites with many patterns aligned (so called ‘hot spots’). The next step is identification of miRNAs that are likely to bind to these sites. This approach also allows to identify sites targeted by yet-undiscovered miRNAs. The minimum number of base-pairs between miRNA and mRNA, the maximum number of unpaired bases and the free energy cutoff are user-defined parameters. Rna22 does not exploit cross-species conservation alignment for final scoring.

## PRACTICAL ASPECTS OF miRNA TARGET PREDICTION ALGORITHMS 

Since the appearance of target prediction algorithms there has been a need to evaluate their precision and authenticity of outcome scores in order to estimate their effectiveness and select the best ones. Target prediction programs are a type of binary classification tests addressing the question of whether there is an interaction between miRNA and given target transcript or not. There are two statistical parameters that could be used to characterize their performance, namely sensitivity, a percentage of correctly predicted targets out of total correct ones, and specificity, a percentage of correctly predicted among overall predicted ones. These parameters are strictly connected with the false positive and false negative rates that, respectively, describe a number of targets that were experimentally rejected as untrue and targets that exist *in vivo* but are not predicted computationally. The choice of a program or programs for the analysis should take these two measures into account. In general, the emphasis is put more on sensitivity in the search of all potential targets for specific miRNA and on specificity in the examination of miRNAs regulating a single gene.

### Comparison of miRNA Target Prediction Algorithms

The first study of target prediction algorithms performance was carried out by Sethupathy *et al.* in 2006 [61]. The specificity and sensitivity were calculated using a set of experimentally validated mammalian targets from TarBase [49]. TargetScanS, PicTar and miRanda used alone or as a union (targets indicated by at least one of these algorithms) made the best tradeoff between sensitivity and specificity while TargetScan and DIANA-microT did not succeed. Also, only the intersection of TargetScanS and PicTar (interactions predicted by both programs) achieved good results which could be explained by exploiting similar strategy for miRNA target prediction based on evolutionary conservation of binding sites and strict complementarity within seed regions. The method of examination proposed by Sethupathy, however, might lead to wrong conclusions. The group of experimentally validated targets was not large and, more importantly, it consisted of only such interactions that had been discovered with the usage of the predetermined set of rules for miRNA target prediction. Because of still not having defined all possible types of miRNA-mRNA interaction, the analyzed group could not be regarded as representative.

High throughput methods provided information about the miRNA effects at the proteome level. The whole context of miRNA regulation was analyzed and therefore miRNA target prediction algorithms were compared more precisely. Baek *et al.* in 2008 [62] applied a quantitative-mass-spectrometry-based approach using SILAC (stable isotope labeling with amino acids in cell culture) to study the influence of miR-223 on the protein output in mouse neutrophils. The comparison between *in vivo* results and predictions *in silico* revealed that TargetScan and PicTar seemed to be the best ones and moreover, only TargetScan total context score, that is assigned to each result, correlated with protein downregulation. Algorithms not using evolutionary analysis such as PITA should be rather used for non-conserved targets only and still need an improvement. Analogous research was carried out by Alexiou *et al.* in 2009 [63]. They used data obtained *inter alia* from Selbach *et al.* experiments [64] that consisted in employing a modified SILAC method (pulse-labeling with two different heavy isotopes) combined with mass-spectroscopy analysis to observe changes in protein production after overexpression of 5 miRNAs (miR-1, miR-16, miR-30a, miR-155 and let-7b) in HeLa cells. The results showed that five programs (TargetScan, TargetScanS, PicTar, DIANA-microT and EIMMO [29]) had a specificity of around fifty per cent and six to twelve per cent of sensitivity which correlates with other reports [65]. Furthermore, they conducted similar analysis for all unions and intersections of miRNA target prediction algorithms. It can be concluded that a target predicted by more than one program is more likely to be true than other targets, although such an intuitive tendency may be misleading in many cases. The combinations of algorithms may result in the increase of the sensitivity at the cost of specificity.

In sum, there is no universal miRNA target prediction algorithm that can be used routinely and efficiently for every 3’UTR sequence since not all rules of mRNA-miRNA interactions have been discovered yet. Therefore, instead of having a clear result of computational analysis specifying whether there is a functional binding site or not, target prediction programs give point scores and percentages that only assess the possibility of interaction. This is a reason why researches from other fields of biomedical sciences are often lost in the abundance of available information and variety of miRNA target prediction algorithms. 

### Practical Insight into miRNA Target Prediction

**Fig. (2). The flow chart of steps for efficient miRNA target prediction.**
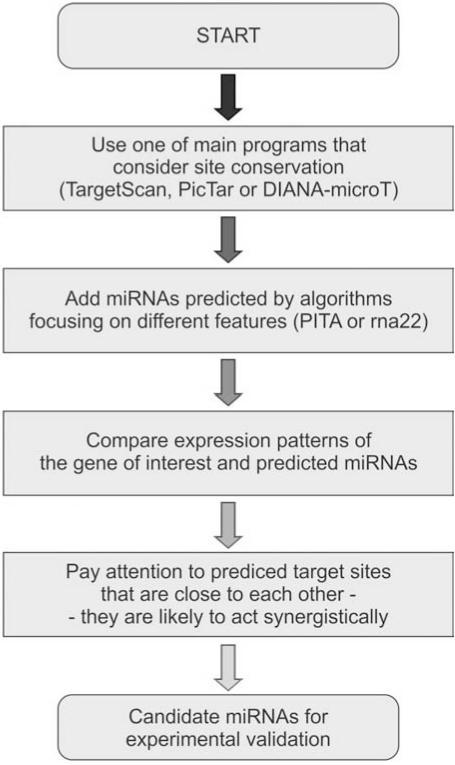
Details discussed in the main text.

To meet the need of a guidance in miRNA target prediction, the information about distinctive features of commonly used algorithms and their performance, advantages and drawbacks has been gathered (Table **1**) and a flow chart showing the stages of the miRNA target prediction has been shown (Fig. **2**). Table **1** presents the main parameters that are considered by programs for final scoring and also provides information about the conservation, which is the main feature distinguishing miRNA target prediction algorithms. Some of programs use the cross-species alignment as an indispensable factor to filter out false negatives (e.g., PicTar) or as a user-defined parameter to reduce the number of putative target sites (PITA), while other algorithms do not exploit this feature for final scoring (rna22). In many cases, the basic concept employed by algorithms in search of miRNA-mRNA predetermines the outcome advantages and weak points of used strategies. For instance, miRanda allows a wobble pairing within the seed region which adds 3’ compensatory sites to the list of predicted targets. However, at the same time, the allowance of wobble pairing lowers the algorithm precision. DIANA-microT examines each target site independently, providing additional parameters such as SNR and the probability of being a true site. Therefore DIANA-microT does not favor miRNAs with multiple target sites. PITA and rna22 are examples of algorithms focusing on novel features of miRNA-mRNA interaction; target site accessibility and pattern recognition, respectively. Their use broaden the list of potential miRNA target sites but these sites are predicted with low efficiency.

The most suitable approach for miRNA target prediction seems to be dependent on the nature of planned experiments. Basic scientists want to perform comprehensive analysis to discover all true interactions while clinically-orientated researchers may be satisfied with the strongest interactions that could be employed in gene therapies. In Fig. (**2**) we present a schematic procedure which we believe will help to choose the appropriate strategy for computational analysis of miRNA-mRNA interactions. The first step is to use one of the programs that consider site conservation (PicTar, TargetScan or DIANA-microT) because they are characterized by high precision and sensitivity. As it was mentioned before, the combined use of them as an intersection or a union is less effective in most cases and therefore this procedure should not be used. These algorithms cannot be used for newly evolved genes that do not have their orthologs in distantly related species. The next step is to add targets indicated by programs exploiting other parameters for final scoring (e.g. PITA, rna22). This action should be treated as optional and it is encouraged to follow especially if previous steps give few putative miRNA-mRNA interactions. After selection of predicted miRNAs regulating the gene of interest, the expression profile of miRNAs and the gene should be compared to detect the overlaps and/or inverse correlations. miRGator, a tool for an integration of miRNA and mRNA expression data have been created [66]. It is a crucial step for assessing the putative physiological regulation of gene by miRNAs, especially when tissue-specific expression of miRNA may be linked to the disease of interest. Finally, close attention should be paid to putative target sites that are located in the immediate vicinity. Both the genome-wide analysis [33] and the experimental data [35] demonstrated that sites located close to each other often act synergistically. Although this feature is still not included in miRNA target prediction algorithms, it may play an important role in the mechanism of miRNA-mediated gene regulation.

## EXPERIMENTAL VALIDATION OF PREDICTED TARGET SITES

Once miRNA has bound to its binding site within 3’UTR of the targeted transcript the following biological effects may be exerted: translational arrest effecting in protein level decrease, deadenylation of the transcript and/or its degradation resulting in both mRNA and protein levels reduction [4, 5]. The most straightforward method for verification of miRNA function is a transfection of cells with miRNA mimetics or miRNA inhibitors, followed by quantitative analyses of target mRNA and protein levels [67, 68]. miRNA mimetics imitating endogenous miRNAs may be delivered either in the form of synthetic siRNA-like duplexes or miRNA-encoding vectors (recently reviewed in [69]). Specific miRNA inhibitors commonly used to silence miRNA function are complementary oligoribonucleotides, usually modified, such as 2’-O-methyl-modified oligoribonucleotides [70], LNAs (locked nucleic acids) [71] and antagomirs (cholesterol-conjugated single-stranded RNAs) [72], or vector-based transcripts called “miRNA sponges”, containing multiple miRNA binding sites that absorb miRNAs and prevent binding their targets [73, 74]. To confirm direct interactions between miRNAs and mRNAs comprehensive analyses should be performed. Several methods for experimental verification of predicted miRNA-mRNA interactions are currently being used and percentage shares of targets validated by the use of a particular method are presented in Fig. (**3**). The most commonly used methods, i.e., reporter assays, microarrays and proteome analyses are detailed below. Experimentally validated miRNA-mRNA interactions have been gathered in various databases, such as TarBase [50], MiRecords [75], Ago [76] and miRNAMAP [77].

### Gene Reporter Assays

**Fig. (3). The pie chart showing percentage shares of methods used successfully to validate experimentally miRNA targets.**
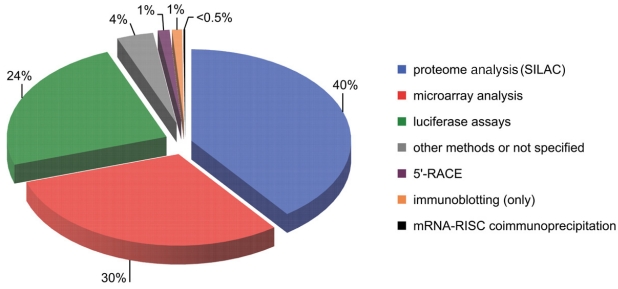
The numbers show the percentage shares of targets validated by the use of a particular method. Data were obtained from the latest release of Tarbase v 5.0, June 2008 [50].

The very first approach exploited in the field of experimental validation of putative miRNA-mRNA interactions was the use of reporter assays, usually luciferase reporter assays, because miRNA activity on such reporter genes can be easily measured [42, 58]. This method is based on cloning 3’UTRs of genes of interest or 3’UTR segments containing miRNA binding site(s) into expression vectors bearing a reporter gene. As a negative control, constructs carrying 3’UTRs with mutated target sites that unable miRNAs binding are also used [36, 40, 78-82]. Transient transfection of cells with reporter constructs followed by measurement of the reporter activity enables validation of predicted miRNA-mRNA interactions. Cells not expressing a miRNA of interest may be co-transfected with the reporter construct and either a miRNA mimetic or a miRNA-encoding vector. This method is frequently extended and the miRNA-mRNA interaction is further confirmed by a transfection with miRNA inhibitors. The reporter assay still serves as an efficient and routinely used strategy for the verification of individual miRNA-mRNA interactions [83] (Fig. **3**). Simplicity of the method is its main advantage, while the fact that it does not allow a high-throughput identification of miRNA targets is an important drawback.

### Microarray Analysis

An increasingly popular and high-throughput method used in the experimental validation of miRNA-mRNA interactions is microarray analysis [65, 84] which takes advantage of the fact that one of the direct effects of miRNA binding is a simultaneous reduction in targeted transcripts’ levels [5, 62]. Such an analysis consists in the comparison of cell transcriptomes after miRNA overexpression or knockdown with reference to the transcriptome of untreated cells. The significant advantage of this method ids the fact that it enables performing a large-scale analysis. However, not all miRNA-mRNA interactions may be discovered by this method since miRNAs can downregulate genes by lowering protein expression levels with an undetected change in transcript levels [5]. Moreover, the transcriptomes vary between different tissues and highly depend on the cell physiology. Therefore, a strategy involving multiple set of microarrays was proposed to overcome these problems and ensure reliability of the method [85].

### Proteome Analysis

Another high-throughput method successfully used in validation of miRNA target sites is the proteome analysis [62, 64]. Similarly to microarrays, the proteomics approach is based on measuring the change of protein level in response to miRNA introduction. This method employs stable isotope labeling with amino acids in cell culture (SILAC) followed by a quantitative-mass-spectrometry [62, 86]. Later, this technique was modified by conducting pulse labeling with two different heavy isotopes [64]. The proteome analysis is considered to be more proper to detect functional miRNA-mRNA interactions than microarrays but still both methods share common limitations, such as high dependency of trancriptome and proteome on cell physiology. Moreover, certain changes detected in protein levels may result from an indirect miRNA regulation instead of a direct effect of miRNAs binding to the targeted transcripts which is also a disadvantageous feature of the method.

### Immunoprecipitation and Other Methods

Apart from the strategies mentioned above, other methods for experimental validation of miRNA-mRNA interactions have been introduced and successfully used (recently reviewed in [87]). AGO proteins of RISC can bind both miRNAs and mRNAs and this feature was exploited in co-immunoprecipitation assays [88, 89]. Used collectively with deep sequencing, AGO immunoprecipitation allowed validating of miRNA-mRNA interactions in a genome-wide manner. HITS-CLIP, namely high-throughput sequencing of RNAs isolated by crosslinking immunoprecipitation, was used to directly identify AGO-bound miRNAs and their target mRNAs in the mouse brain [90]. Such an approach complemented the bioinformatic way of the miRNA target identification and reduced the number of false-positive predictions. More recently, CLIP method was modified in respect of an ultraviolet irradiation used to covalently crosslink RNA-protein complexes within cells. The improved CLIP, termed PAR-CLIP (Photo-activatable-Ribonucleoside-Enhanced Crosslinking and Immunoprecipitation) was used by Hafner *et al*. [91] to analyze miRNPs (miRNA-RNA-protein) complexes in HEK293 cells. Despite being regarded as a very modern and elegant way to perform large-scale analyses, CLIP methods have some weaknesses. Not only are they technically challenging and expensive but frequently encounter problems with distinguishing between direct and indirect miRNA-mRNA interactions. 

Furthermore, methods based on different approaches were also successfully implemented for the verification of miRNA-mRNA interactions. Davis *et al.* [92] conducted RNA-ligase-mediated (RLM) 5’ RACE experiments to verify miRNA-target interactions, and Li *et al.* [93] proposed that high cytoplasmic-to-nucleic ratio of mRNA expression may indicate genes that are likely to be controlled by miRNAs. In the latter report it was suggested that a measurement of the transcript level by a microarray or fluorescent real time PCR (qPCR) preceded by a computational analysis can be performed to create a list of potential targets [93]. 

### Evaluation of miRNA Target Validation Methods

Commonly used methods for experimental validation have their own assumptions, strengths and weaknesses (reviewed in [94]). The most promising techniques are those which may be converted to a multiplex format, however they are not fully satisfying in terms of their sensitivity. On the other hand, more sensitive methods (e.g., reporter assays) cannot be used in large-scale analyses. Certain targets validated by reporter gene assays may be also false negatives as an effect of RISC saturation due to miRNA overexpression. Furthermore, inhibitors designed to exclusively repress the function of the specific miRNA (frequently used in all validation methods) may not be selective enough since many miRNAs belong to miRNA families with common seed regions and such inhibitors could trigger non-specific repression in many cases. Taken together, there are many pitfalls associated with miRNA target validation. All validation methods, including their advantages and drawbacks as well as future perspectives of miRNA target identification and miRNA-mRNA interaction analyses, have been extensively discussed elsewhere [54, 87, 94-96].

## PREDICTION OF miRNA TARGETS IN TREDs

Many studies showed that miRNAs play a crucial role in the development and functional regulation of nervous system and deregulation of miRNAs was postulated and proved in various neurological disorders [97-100]. There have been published several reviews summarizing our current knowledge of neurodegeneration and its connections with miRNA regulation [101-105] but have been rather focused on neurodegenerative diseases such as Alzheimer’s and Parkinson’s. In this section we have summarized current knowledge about experimentally proven associations between miRNAs and TREDs (Table **2**). Moreover, we provide a detailed analysis and show the practical side of miRNA target prediction on the example of miRNA interactions with mRNAs derived from genes triggering the pathogenesis of trinucleotide repeat expansion diseases (TREDs).

### Brief Characteristics of TREDs and Mechanisms of Pathogenesis

TREDs comprise mostly neurodegenerative disorders, such as Huntington’s disease (HD) and a number of spinocerebellar ataxias (SCAs), and neuromuscular disorders, such as myotonic dystrophy type 1 (DM1). The underlying cause of TREDs is an expansion, to an abnormal length, of trinucleotide repeats (TNRs), CAG, CTG, CGG and GAA, that occur both in coding and non-coding regions of human genes [106-108]. The same type of mutation is a characteristic of all genes triggering TREDs but these genes do not perform similar functions. However, this group of disorders could be divided into categories with regard to the localization of expanded TNRs within genes and the mechanism of pathogenesis. The expansion of TNRs could result in either the loss-of-function mechanism which consists in protein function impairment, i.e., reduction or absence of protein production, or the gain-of-function resulting in an altered function of the mutant protein or toxicity of the mutant transcript. The mechanism of pathogenesis also determines the choice of miRNA features that should be considered in the employment for therapy. 

### microRNAs as Therapeutic Agents

Polyglutamine (Poly-Q) diseases, a group of TREDs such as HD and several SCAs where the translated CAG repeats expand, are predominantly caused by the toxic polyQ-expanded protein [106-108] with possible contribution from mutant transcript [109, 110]. As yet no conventional therapy could be applied to treat patients with these diseases and employment of miRNA machinery and function seems to be one of the promising therapeutic approaches. Pioneering research in this area was conducted by Lee *et al.* [111]. They showed that the level of ATXN1, the protein being a product of *ATXN1* gene, is modulated by miRNAs and the inhibition of miRNAs targeting ATXN1 transcript enhanced cell toxicity. In the case of polyQ-diseases, miRNAs could reduce levels of both the affected protein and mutant transcripts. Although miRNAs would not discriminate between the mutant and normal transcripts, tighter regulation of the protein level itself was demonstrated to have therapeutic efficacy in HD [112]. Another possibility to consider is to construct artificial miRNAs targeting new sites in 3’UTRs. Taking advantage of miRNAs as therapeutic agents may be more beneficial in some circumstances than the use of siRNAs since miRNAs could offer better specificity and lower toxicity [20]. It appears to be prudent to deliver to the affected tissue lower doses of different specific miRNAs instead of a high dose of single miRNA regulating the gene of interest, since the effect of lower doses should be additive. This approach may also diminish the undesired effect of decreasing the off-target genes’ expression because the most downregulated would be genes having target sites for all introduced miRNAs. An interesting approach to explore would be a use of miRNAs that regulate the gene of interest but are not expressed in the affected tissues. This strategy could provide higher specificity since a smaller number of existing transcripts may have target sites for the miRNA not expressed normally in this tissue.

DM1 is caused by the accumulation of a toxic transcript in the nucleus [113] which apparently limits the use of miRNAs in the therapy since miRNA regulation takes place predominantly in the cytoplasm. The transcript toxicity would narrow down miRNAs to those that act as gene regulators both on the protein and the transcript levels but this does not seem to be a serious limitation as it was proved recently that majority of miRNAs decrease target mRNA levels [5]. Furthermore, it was shown that miRNAs having nuclear localization signal were imported to the nucleus and downregulated nuclear transcripts [114]. It may be beneficial to employ this knowledge in constructing artificial miRNAs for targeting the DMPK transcript retained in the nucleus. The possibility of targeting genes in an allele-specific manner in the disease caused by expanded CUG repeats such as DM1 was also considered as miRNAs with CAG repeats in their seed regions may potentially regulate mutated alleles more tightly. It was proposed in Hon and Zhang [28] that the length of CUG repeats could correlate with the miRNA repression in the case of 3’UTR of DMPK. Six miRNAs having CAG repeats within their seed regions were identified and it was indicated by *in silico* studies that they could regulate the expression of *DMPK* by multiple targeting the region of CTG repeats in its 3’UTR. Further analysis and experimental validation of this proposition is needed.

### Analysis of miRNA-mRNA Interactions on the Example of TREDs’ Triggers

**Fig. (4). The graphical presentation of miRNA target sites distribution in 3’UTRs of TREDs genes predicted by selected algorithms.**
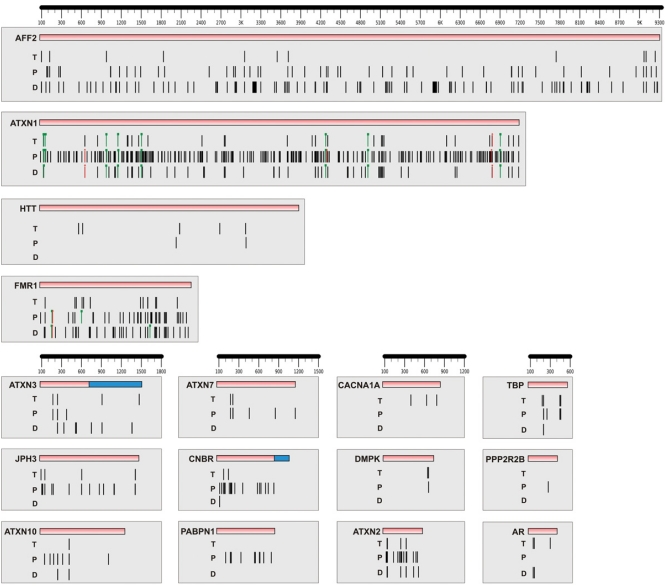
The following algorithms were used for miRNA target prediction: TargetScanHuman Release 5.1 (only conserved sites for miRNA families broadly conserved among vertebrates were considered) (T), PicTar (Lall *et al.* 2006) (P) and DIANA-microT version 3.0 (D). A vertical bar depicts a single seed region of a miRNA target site predicted by the particular algorithm; putative target sites are marked by black vertical bars, experimentally validated by green rectangular-headed and false positives (experimentally rejected as untrue) by red star-headed bars. The absence of bars indicates that the algorithm did not predict any miRNAs targeting specified transcript or the transcript was not included in the UTR base used by this algorithm. The horizontal black lines with the division are scales with base pairs as a unit of measure. 3’UTRs of TREDs genes are grouped on the basis of their length. The sequence of 3’UTR that is common for all three algorithms is colored in pink. In the case of ATXN3 and CNBP 3’UTRs, the blue color indicates regions not analyzed by PicTar due to different criteria used to define 3’ UTR boundaries. No target sites were found for *ATN1* and *FXN* genes. 3’UTR of *ATXN8(OS)* gene is not identified. The images of 3’UTRs with miRNA distribution were created using FancyGene v 1.4 [128].

In our analysis, we have focused on searching for miRNAs directly regulating genes causing TREDs by exploiting three commonly used algorithms (TargetScan, PicTar and DIANA-microT) for the target prediction. We combined the results obtained by these algorithms and showed graphically the distribution of putative miRNA binding sites in 3’UTRs (Fig. **4**). The detailed list of all identified miRNAs and interaction parameters is presented as supplementary data. 

Since both the number of miRNA sites and their arrangement influence the degree and specificity of miRNA-mediated gene repression [28] it is of utmost importance to carefully analyze the distribution of miRNA binding sites throughout the whole 3’UTR. Close attention should be paid to the following: the 3’UTR length, density of miRNA binding sites, existence of multiple sites and the distance between them (see the paragraph *“miRNA-mRNA interactions”*). We mapped miRNA recognition sites for 16 genes involved in the pathogenesis of TREDs and the outcomes of three algorithms were shown collectively one below the other as a triplicate-like analysis. Such a graphical presentation facilitates swift and correct interpretation of the results and therefore may help to choose miRNA-mRNA interactions for experimental validation. Additionally, we presented experimentally verified target sites found in the case of ATXN1 and FMR1 3’UTRs; the sites which were confirmed to be true are marked by green and rejected as untrue by red color. The analyzed TREDs-related genes varied significantly in their 3’UTR lengths, ranging from 436 nt in the case of *AR* gene to 9330 nt for *AFF2* gene. 3’UTR lengths did not correlate with the number of miRNA binding sites. The *HTT* gene was predicted to have only a few sites for miRNA-mRNA interactions despite its significant length (3900 nt) while an abundance of putative miRNA sites was found for a relatively short (596 nt) *ATXN2* gene. There were 7 and 19 binding sites in HTT and ATXN2 3’UTRs, respectively. Moreover, neither the number of miRNA sites nor their location in 3’UTR was readily comparable between results obtained by three algorithms. Although we chose programs that are based on sequence conservation alignment, their outcomes overlapped only to a limited extent. It is justifiable and seems to be a consequence of putting emphasis on different parameters for final scoring. 

Our target prediction analysis served as a point of reference to discuss the recent achievements in the field of searching for links between miRNA and TREDs. Lee *et al.* [111] exploited PicTar for target prediction analysis of *ATXN1* gene and showed its downregulation by three miRNAs while two out of three having multiple biding sites within 3’UTR of ATXN1 mRNA. Eight predicted miRNAs were chosen for the experimental analysis while PicTar predicts a lot more miRNAs being likely to associate with 3’UTR of ATXN1 (Fig. **4**). PicTar indicated binding sites which are equally distributed within 3’UTR whereas TargetScan and DIANA-microT predicted fewer binding sites but they coincided to a high degree. Interestingly, all experimentally confirmed target sites were detected by each used algorithm which proved that approaches used by algorithm designers seem to be similarly efficient. One target site which turned out to be a false negative was detected only by PicTar, the second one both by DIANA-microT and PicTar while the third one by all algorithms. Although the minority of putative target sites were selected for validation, Lee *et al.* analyzed them thoroughly. miRNAs of chosen target sites were examined by miRNA duplex transfection and by the use of specific 2’-O-methyl inhibitors followed by measurement of the level of protein (western blot) and RNA decrease (RT-PCR). Exact validation of chosen target sites was carried out by using whole or parts of *ATXN1* 3’UTR fused with firefly luciferase reporter genes and by mutagenesis of putative sites. Furthermore, the coexpression of ATXN1 and specific miRNAs were shown by conducting a northern blot analysis and RNA in-situ hybridization. Such complete analysis meets the criteria elaborated by Kuhn *et al.* [95] to validated target sites as confirmed. Sinha *et al.* [115] demonstrated that gene encoding TATA-binding protein (TBP), which is mutated in SCA type 17 (SCA17), is downregulated by miR-146a. It was predicted by miRanda and RNAHybrid but none of the three algorithms used in our analysis managed to predict this interaction. This is another proof that miRNA target prediction still needs further modification and it is highly likely that all true miRNA interactions may not be predicted by using only one, two or even three algorithms. Recently, it has been demonstrated that three miRNAs interact with binding sites in the 3’UTR of FMR1 mRNA [116]. Yi *et al.* created constructs expressing a firefly luciferase with native *FMR1* 3’UTR and 3’UTR with mutated putative target sites and constructs of pre-miRNAs fused with green fluorescent protein (GFP). More extensive research has to be conducted to examine the impact of miRNAs downregulating the 3’UTR of *FMR1* on its transcript and protein levels. The first of the positively validated sites were predicted both by PicTar and DIANA, the second one only by DIANA-microT and the third one exclusively by PicTar. The first site was also indicated by TargetScan with the annotation that this site is conservative but the potential miRNA belongs to the non-conservative family among mammals. Moreover, one of the rejected target site as untrue were not detected by neither of three target prediction programs. Other TREDs genes were not investigated for their direct regulation by miRNAs. Our computational analysis clearly shows that some of them, most of all *AFF2* and *ATXN2*, may have multiple functional binding sites for miRNAs (Fig. **4**) which are worthy for further examination and experimental validation. On the other hand, TBP has a short 3’UTR with only a few possible binding sites which is consistent with the results of the comprehensive analysis of the length and sequence of 3’UTRs of different genes. Taken together, this analysis showed that among TREDs’ triggers there are genes that are tightly regulated by miRNAs and genes involved in basic functions such as housekeeping genes, avoiding miRNA sites which observation has an evolutional explanation [117].

### Studies of miRNA Deregulation in TREDs 

Another approach to exploit miRNA regulation in TREDs studies for a better understanding the mechanism of neurodegeneration and a possible future clinical application was to compare miRNA expression levels between non-affected and affected individuals. Studies of *D. melanogaster* showed miRNAs are involved in modulation of neuron survival in response to the degeneration caused by polyglutaminate (polyQ) tracts [118, 119]. *Ban*, a fruit fly miRNA involved in tissue growth and programmed cell death [120], plays a protective role in polyQ-induced degeneration. Although there is no orthologue of *ban* in human cells, this regulation suggests that miRNAs can participate in TREDs pathogenesis by regulating cell degeneration or by being affected by mutant proteins  or transcripts. Furthermore, many links between miRNA pathways and *FMR1*, the gene mutated in fragile X syndrome (FXS), were discovered [121, 122]. FMRP, the protein product of *FMR1* gene, was proved to interact with key RISC proteins such as AGO1, AGO2 and Dicer and associate with endogenous miRNAs [123, 124]. Moreover, FMRP plays role in processing of pre-miRNAs [125, 126]. Several studies [92-94] proved that mutant huntingtin, a product of the *HTT* gene, is incapable of associating with Repressor element 1-silencing transcription factor (REST) which leads to repression of neural miRNAs and their lower levels in the brains of HD patients. Recent publications show miRNA deregulation in HD may be more extensive [86]. Marti *et al.* [127] used next generation sequencing (NGS) methods to identify the changes of miRNAs composition and their expression levels in patient with HD with reference to non-affected individuals. Moreover, significant changes in miRNA editing have been observed. Prediction and validation of genes targeted by miRNAs that are altered in HD patients is important for better understanding of pathogenic mechanisms leading to neurons death.

## CONCLUDING REMARKS 

Many computational tools have been designed for miRNA target prediction but may frequently lead to growing confusion. There is no single algorithm that can be used routinely for every analysis of 3’UTR sequences. Gaining more and more knowledge about miRNAs and their role in gene regulation prompted researchers to revise the way of analyzing individual miRNA-mRNA interactions. It is recommended to see miRNA regulation as a complex network involving genes targeted by many miRNAs and often having multiple sites for the same miRNA [94]. The outcome of such interactions is context-dependent and therefore it is difficult to reconstruct reliable miRNA-mRNA interactions in experiments conducted* in vitro*. Moreover, it is hard to assess to which extent the transcript level should be repressed to consider miRNA-mRNA interaction is truly functional.

On the strength of our current knowledge of miRNA-mRNA interactions we propose to follow a few guidelines for miRNA target prediction (Fig. **1**). They originate in research studies considered in this paper and our discussions. We advise to use first miRNA target prediction algorithms focusing on the orthologous sequence alignment and then possibly apply algorithms considering other parameters such as free energy of binding or target site accessibility. The inverse correlation between expression levels of putative miRNA and targeted mRNA and/or protein levels increases the likelihood that this interaction is real and functional. Expression data can be obtained from microarray or proteomics databases or with the use of miRGator [66]. We would like to draw attention to the possibility of mutual interactions between miRNA sites located close to each other. We hope these conclusions will be beneficial for researches from other fields of biomedical sciences willing to implement the search for miRNAs in their molecular studies.

## SUPPLEMENTARY MATERIAL

Supplementary material is available on the publishers Web site along with the published article.

## Figures and Tables

**Table 1:** Features, Experimental Evaluation Results and Assessment of Commonly Used Algorithms in miRNA Target Prediction

Target prediction algorithm	Features		Experimental evaluation results				Assessment		Reference
	Parameters contributing to the final score	Cross-species conservation	Sethupathy *et al.* 2006	Baek *et al.* 2008	Alexiou *et al.* 2009		Advantages	Disadvantages	
			sensitivity^1^	log_2_-fold change^2^	precision^3^	sensitivity^4^			
**miRanda**	complementarity and free energy binding	conservation filter is used	49%	0.14	29%	20%	- beneficial for prediction sites with imperfect binding within seed region	- low precision, too many false positives	[56]
**TargetScan**	seed match, 3’ complementarity local AU content and position contribution^5^	given scoring for each result	21%	0.32^6^	51%	12%	- many parameters included in target scoring - final score correlates with protein downregulation	- sites with poor seed pairing are omitted	[38]
**TargetScanS**	seed match type	only conservative sites are considered	48%	–	49%	8%	- simple tool for search of conserved sites with stringent seed pairing	- underestimate miRNAs with multiple target sites	[38]
**PicTar**	binding energy, complementarity and conservation	required pairing at conserved positions	48%	0.26	49%	10%	- miRNAs with multiple alignments are favored	- does not predict non-conservative sites	[42, 57]
**DIANA-microT**	free energy binding and complementarity	dataset of conserved UTRs among human and mouse is used	10%	–	48%	12%	- SNR ratio and probability given for each target site - possibility of using own miRNA sequence as an input	- some miRNAs with multiple target sites may be omitted	[36]
**PITA**	target site accessibility energy	user-defined cut-off level	–	0.04^6^	26%	6%	- the secondary structure of 3’UTR is considered for miRNA interaction	- low efficiency compared to other algorithms	[46]
**Rna22**	pattern recognition and folding energy	not included	–	0.09	24%	6%	- allows to identify sites targeted by yet-undiscovered miRNAs	- low efficiency compared to other algorithms	[58]

1 percentage of experimentally supported miRNA-target gene interactions predicted (used TarBase records for which a direct miRNA effect was examined).

2 average protein depression of genes predicted by the algorithm to be miR-223 targets.

3 proportion of correctly predicted target miRNAs to total predicted miRNA-mRNA interactions (data obtained from proteomic analyses carried out by Selbach *et al.*).

4 proportion of correctly predicted target miRNAs to total correct miRNA-mRNA interactions (data obtained from proteomic analyses carried out by Selbach *et al.*).

5 *position contribution* parameter promotes sites close to the 3’UTR ends.

6 the final scoring correlates with the level of protein downregulation.

**Table 2:** The Summary of Current Examinations of Links Between miRNA Regulation and TREDs

Disease	miRNA change/regulation	miRNA target prediction algorithms	Methods used for experimental validation	Experimental models	References
Spinocerebellar ataxia type 1 (SCA1)	miR-19, -101 and -130a downregulate *ATXN1* gene	PicTar was used to computationally predict miRNAs targeting ATXN1 transcript. Eight most likely miRNAs were chosen for experimental validation	transfection with miRNA duplexes and their specific inhibitors followed by western blot analysis and RT-PCR	MCF7, HEK293T, NIH3T3 and HeLa cell lines	[111]
			luciferase reporter assays with vectors carrying 3’UTR fragments or mutated target sites	HeLa cell line	
			miRNA detection by northern blot analysis and* in situ* hybridization of mouse RNAs derived from cerebellum	C57/B6 WT mouse	
			cell death assays with mutant ATXN1deprived of target sites	HEK293T cell line	
Spinocerebellar ataxia type 3 (SCA3) and possibly other polyQ disorders	*ban*, a dma-miRNA, modulates polyQ-toxicity	–	phenotype comparison analysis (mutants)	*D. melanogaster*	[118, 119]
			cell death assays	*D. melanogaster* cell line with *ban* overexpression	
			Dicer downregulation	flies and human cell lines	
Dentatorubral pallidoluysian atrophy (*DRPLA*)	dma-miR-8 downregulates *D. melanogaster *atrophin gene	–	phenotype comparison analysis (mutants)	*D. melanogaster*	[129]
			mutant expression profiling microarray analysis	fruit fly *pupae*	
			real-time RT-PCR with intron specific primers	fruit fly *pupae*	
			luciferase reporter assay with vectors containing 3’UTR with mutated target sites	fruit fly *pupae* and S2 cell line	
			*in vivo* studies of miR-8/atrophin functionality (death assays of mutants with atrophin and/or miR-8 underexpression, immmunocytochemistry for apoptotic cell detection)	*D. melanogaster*, fruit fly embryos	
Huntington’s Disease (HD)	downregulation of miR-9, -9*, -29b, -124 and upregulation of miR-132 associates with HD	–	quantitative real-time PCR (qPCR) using TaqMan miRNA assays	human brain post mortem samples of the Brodmann’s area 4 (BA4) cortex	[130]
			co-transfection of miRNA precursors and REST/CoREST 3’UTRs with luciferase assay followed by western blot analysis	HEK293 cell line	
Huntington’s Disease (HD)	downregulation of miR-132 and upregulation of miR-29a and -330 associate with HD	–	infection with adenovirus expressing a dominant-negative REST construct followed by RT PCR	cell lines of wt and mutant Hdh knock-in embryonic mice	[131, 132]
			qPCR using pre-miRNA stem loop primers	R6/2 mouse (and human) post mortem samples of the cortex (BA4 cortex)	
Huntington’s Disease (HD)	downregulation of 15 miRNAs^1^ and upregulation of 19^2^ and miRNA editing alterations associate with HD	microPred pipeline for novel miRNAs prediction [133], TargetScan for prediction of genes regulated by altered miRNAs	massively parallel sequencing of small non-coding RNAs (ncRNAs) followed by TaqMan microRNA assays	human brain post mortem samples of the frontal cortex (FC) and the striatum (ST)	[127]
Huntington’s Disease (HD)	downregulation of 15 miRNAs^3^ and upregulation of 9^4^ associates with HD	miRNAMap 2.0 resource [77] (miRanda, TargetScan, RNAHybrid) was used for prediction of genes regulated by altered miRNAs	qPCR using stem loop primers	STHdh^Q111^/Hdh^Q111^ cells - cell lines of wt and mutant Hdh knock in embryonic mice	[115]
Spinocerebellar ataxia type 17 (SCA17)	miR-146a downregulates *TBP* gene		luciferase reporter assay with vectors containing exogenous TBP 3’UTR with western blot analysis co-transfection of miRNA precursors and TBP 3’UTR followed by northern blot analysis		
Myotonic dystrophy type 1 (DM1)	overexpression of miR-206 associates with DM1	–	qPCR using TaqMan microRNA assays northern and western blot analysis *in situ* hybridization of miR-206 using locked nucleic acid probes (LNA)	human muscle samples of vastus lateralis	[134]
Fragile X syndrome (*FXS*)	dFmrp associates with RISC and endogenous miRNAs	–	transcfection with vectors containing dFmrp co-immunoprecipitation with RISC proteins and miRNAs, norther and western blot analysis	S2 cell line	[123]
Fragile X syndrome (*FXS*)	dFmrp is required for processing of miR-124a	–	in situ hybridization of dma-miR-124a	*D. melanogaster* embryos	[125]
			immunoprecipitation with miR-124a, norther and western blot analysis qPCR using stem loop primers	transgenic fruit fly *pupae*	
Fragile X syndrome (*FXS*)	miR-19b, -302b* and -323-3p downregulate *FMR1* gene	intersection of computationally predicted targets by miRbase [3], miRanda and miRDB was used for validation	luciferase reporter assays with vectors containing native 3’UTR or with mutated target sites combined with co-transfection of GFP-tagged plasmids expressing pre-miRNAs	HEK293 cell line	[116]

1 miR-95, -124, -128, -127-3p, -139-3p, -181d, -221, -222, -382, -383, -409-5p, -432 , -433, -485-3p and -485-5p.

2 miR-15b, -16, -17, -19b, -20a, -27b, -33b, -92a, -100, -106b, -148b, -151-5p, -193b, -219-2-3p, -219-5p, -363, -451, -486-5p and -887.

3 miR-9, -9*, -100, -125b, -135a, -135b,-138, -146a, -150, -181c, -190, -218, -221, -222 and -338-3p.

4 miR-145, -199-5p, -199-3p, -148a, -127-3p, -200a, -205, -214 and -335-5p.
